# Ten tips on how to prevent falls in dialysis patients

**DOI:** 10.1093/ckj/sfag167

**Published:** 2026-05-23

**Authors:** Nobuyuki Shirai, Masakazu Saitoh, Atsuhiro Tsubaki, Shinichiro Morishita, Sho Kojima, Yutaka Osawa, Suguru Yamamoto

**Affiliations:** Department of Rehabilitation, Niigata Rinko Hospital, Niigata, Japan; Department of Physical Therapy, Faculty of Health Science, Juntendo University, Tokyo, Japan; Institute for Human Movement and Medical Sciences, Niigata University of Health and Welfare, Niigata, Japan; Department of Physical Therapy, School of Health Science, Fukushima Medical University, Fukushima, Japan; Institute for Human Movement and Medical Sciences, Niigata University of Health and Welfare, Niigata, Japan; Internal Medicine, Niigata Rinko Hospital, Niigata, Japan; Division of Clinical Nephrology and Rheumatology, Niigata University Graduate School of Medical and Dental Sciences, Niigata, Japan

**Keywords:** dialysis, fall prevention, falls, frailty, multidisciplinary care

## Abstract

Falls are frequent and clinically significant events among patients undergoing dialysis and are associated with fractures, hospitalization, loss of independence, and increased mortality. Despite their clinical importance, fall prevention in this population remains challenging in routine practice because fall risk arises from a complex interaction between general geriatric factors and dialysis-specific conditions.

This is a narrative review and proposes a conceptual framework that integrates general fall-related factors with dialysis-specific contributors and emphasizes the dynamic nature of fall risk over time. Because fall risk may fluctuate with changes in dialysis conditions, comorbidities, physical function, and activity levels, regular reassessment and longitudinal management are essential. While primarily based on evidence from hemodialysis patients, some aspects are considered applicable to peritoneal dialysis patients as well. Within this framework, we present 10 practical, clinically oriented tips to support fall prevention in patients undergoing dialysis. These tips address systematic risk assessment, monitoring of hemodynamic instability, individualized exercise and physical activity interventions, environmental and behavioral considerations after dialysis sessions, and the importance of multidisciplinary collaboration.

By translating current evidence into actionable clinical guidance, this review aims to support clinicians in integrating fall risk assessment and prevention strategies into routine dialysis care. A structured, multidisciplinary, and continuously adaptive approach may help reduce falls and improve safety and functional outcomes in patients undergoing dialysis.

## INTRODUCTION

Falls in patients undergoing dialysis represent a major clinical challenge, as they are associated with an increased risk of hospitalization, institutionalization, and mortality [1]. Furthermore, studies have shown that hemodialysis (HD) patients are at a higher risk of serious falls that lead to emergency room visits or hospitalization than peritoneal dialysis (PD) patients [2]. On the other hand, non-serious, accidental falls have been shown to occur at a similar frequency in both HD and PD patients [3]. Despite their clinical importance, the assessment and prevention of falls in this population have often been discussed within the framework of conventional geriatric fall models, which may not fully account for the unique characteristics and treatment-related factors specific to dialysis patients. The purpose of this paper was to integrate existing knowledge regarding the risk of falls, primarily in hemodialysis patients, and to position it as a narrative review that presents a practical framework that is applicable in the clinical setting.

Recent studies have highlighted that falls among HD patients are influenced not only by frailty, declines in physical function [4, 5], and psychological factors such as fear of falling (FOF) [6], but also by factors that are insufficiently reflected in traditional functional assessments, including alterations in neuromuscular control during gait [7]. In addition, dialysis-related treatment factors such as intradialytic hypotension (IDH), fatigue, and fluid shifts have been suggested to play a complex and interrelated role in increasing fall risk [8, 9]. These factors may interact with one another, creating a vicious cycle that progressively amplifies vulnerability to falls.

**Figure 1:**
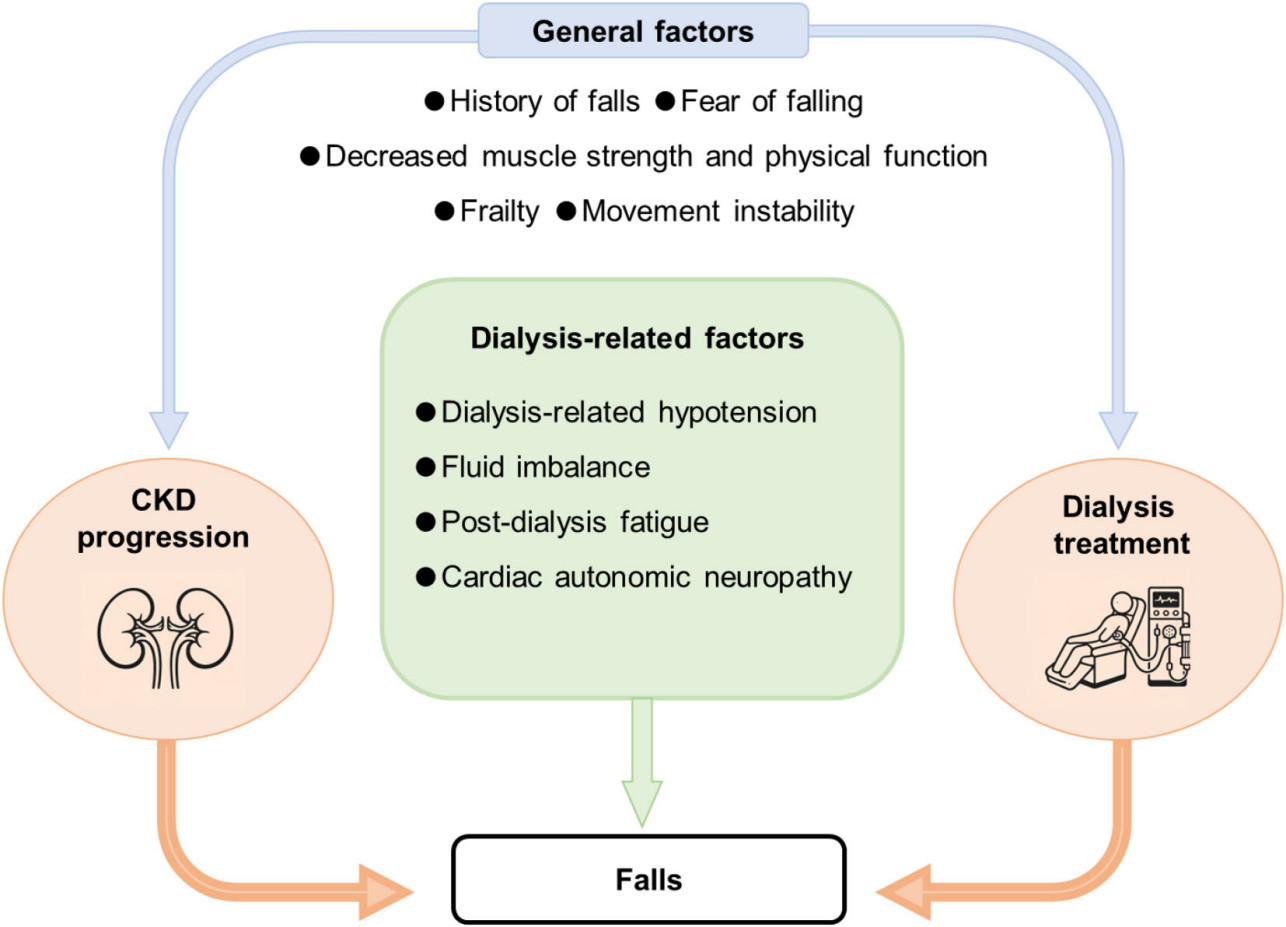
Conceptual model of fall risk in patients undergoing dialysis. This model integrates general risk factors for falls with dialysis-specific factors. These factors interact synergistically and may worsen with CKD progression and dialysis treatment, underscoring the need for comprehensive multidisciplinary prevention strategies.

In this review, we present a conceptual model that organizes dialysis-specific mechanisms contributing to falls (Figure 1) and propose ‘Ten Practical Points for Fall Prevention in Dialysis Patients’ that can be implemented in clinical practice (Table 1). A significant amount of the evidence presented in this paper comes from HD patients; thus, its application to PD patients requires careful interpretation. Adjustments should be made as needed to suit the individual patient’s condition.

**Table 1:** Ten practical tips for fall prevention in dialysis patients.

Tip	Key message	DO	DO NOT	Pitfalls/Limitations
1	Falls should not be considered only a geriatric syndrome	Assess fall risk in all dialysis patients regardless of age	Assume younger patients are at low risk based solely on age or employment status	Younger high-risk patients may be overlooked; current evidence is mainly observational
2	Previous falls are the strongest warning sign	Systematically inquire about fall history and circumstances	Dismiss single or ‘minor’ falls without further evaluation	Falls are often underreported; definitions and recall accuracy may vary
3	Fear of falling is an independent fall risk factor	Explicitly assess fear of falling using questionnaires or structured interviews	Dismiss fear of falling as a personality trait or excessive anxiety	Cut-off values differ across tools; exercise alone may not sufficiently reduce fear
4	Muscle strength and function matter more than muscle mass	Prioritize assessment of muscle strength, gait speed, and balance	Base fall risk assessment solely on muscle mass or sarcopenia diagnosis	Functional tests depend on the setting and assessor; cut-offs are not dialysis-specific
5	Frailty integrates multiple fall risk domains	Use frailty screening tools and intervene early	Consider frailty as irreversible or purely physical	Frailty prevalence varies by tool; transient frailty may be overestimated
6	Falls are multifactorial	Assess cognition, vision, neuropathy, depression, and environment.	Do not rely only on physical function.	Requires resources; interpretation is complex.
7	Dialysis-related hemodynamic changes increase fall risk	Monitor blood pressure changes and post-dialysis symptoms	Treat falls as accidental events unrelated to dialysis treatment	Blood pressure alone may miss autonomic dysfunction; causality is complex
8	Exercise therapy is central, but must be individualized	Provide tailored exercise programs with appropriate safety monitoring	Prescribe uniform high-intensity exercise for all patients	Initial safety concerns exist in high-risk patients; adherence remains challenging
9	Fall risk is dynamic, not static	Perform regular reassessment and adjust interventions accordingly	Rely solely on a single baseline assessment	Optimal reassessment intervals are unclear; resources and staffing may limit implementation
10	Multidisciplinary collaboration is essential	Promote ongoing teamwork across disciplines	Delegate fall prevention to a single profession	Implementation varies across facilities; supporting evidence is still emerging

### Tip 1: Do not label falls solely as a ‘geriatric syndrome’

Falls in HD patients are not limited to older adults. Unlike community-dwelling populations where fall risk typically increases with age, dialysis patients exhibit a high incidence of falls even at younger ages [10, 11]. A recent systematic review and meta-analysis reported a 1-year fall prevalence of 28.8% [10], with recurrent falls (Two or more falls in the past 12 months) [12] occurring in 14.8% of cases [10]. Chronic kidney disease (CKD) progression and dialysis therapy may accelerate biological aging [13], which is more strongly associated with adverse health outcomes than chronological age [14]. For assessing biological age, it is practical to use blood biochemical indicators [15] and functional indicators as surrogate indicators, such as walking speed and grip strength [16]. Taken together, these findings highlight the importance of adopting an approach to assessing fall risk that considers biological rather than chronological age, encompassing all dialysis patients.

### Do

Conduct fall risk assessment for all dialysis patients, regardless of age. Particular attention should be paid to changes in physical function, gait ability, and fall history, especially following hospitalization or the development of additional comorbidities.

### Do not

Do not assume a low risk of falls based solely on demographic factors such as younger age or current employment status. Likewise, avoid applying fall risk assessment tools developed for older adults without considering dialysis-specific factors.

### Pitfalls/limitations

Screening strategies based primarily on age thresholds may not adequately identify younger dialysis patients at high risk of falls. In addition, current evidence regarding falls in younger dialysis patients is largely derived from observational studies; therefore, caution is warranted when interpreting causal relationships.

### Tip 2: A history of falls is the most important warning sign

A history of falls is one of the strongest predictors of future falls in patients undergoing HD [17]. Even a single fall episode may indicate the presence of multiple underlying risk factors in this population. Because older adults often do not spontaneously report falls, it is strongly recommended that healthcare professionals proactively and regularly inquire about fall events [12]. In practice, it is preferable to systematically check the patient’s condition during regular assessments (e.g., monthly or during outpatient visits). Furthermore, for high-risk patients, it may be better to increase the frequency of assessments, for example, to every two weeks, or even more often, if necessary. At a minimum, all patients should be screened annually for falls within the past 12 months, including details of the circumstances [12]. Systematic and repeated assessment of fall history, without trivializing its importance, represents a critical first step in effective fall prevention [12].

### Do

Systematically obtain a history of falls during intradialytic interviews and routine clinical assessments. In addition to the number of falls, clinicians should assess the timing (non-dialysis days, pre-dialysis, or post-dialysis), location (indoors, outdoors, or within healthcare facilities), activity at the time of the fall (e.g., walking, negotiating steps, or standing up), and outcomes (presence of injury or fracture). This detailed information helps clarify the direction and priorities of preventive interventions.

### Do not

Do not dismiss falls as minor events based on explanations such as ‘just a trip’ or the absence of major injury. Likewise, avoid relying solely on observation without conducting appropriate post-fall assessment and intervention.

### Pitfalls/limitations

Patients may underreport falls or inaccurately recall past events. In addition, ambiguity in the definition of a ‘fall’ may result in variability in assessment and documentation across clinicians and care settings.

### Tip 3: Fear of falling is an independent risk factor for falls

**Figure 2:**
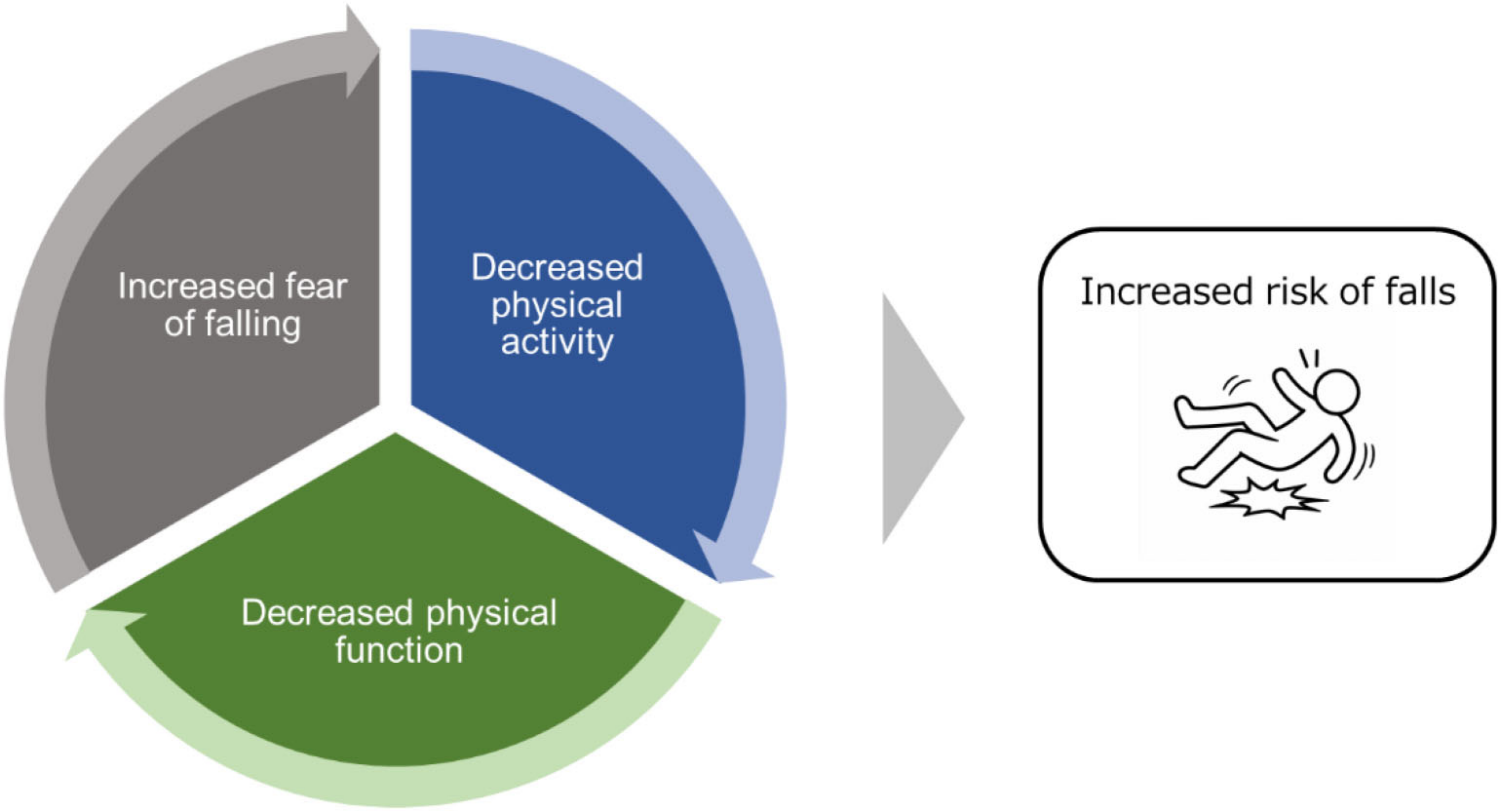
Conceptual model of the vicious cycle contributing to fall risk in patients undergoing hemodialysis. Fear of falling reduces physical activity, leading to functional decline, which further intensifies fear, creating a self-reinforcing cycle. This model illustrates key interrelated domains rather than specific causal pathways.

An increased level of FOF in HD patients is significantly associated with physical function and physical activity and independently predicts future falls, even after accounting for other fall-related risk factors [6]. FOF is a psychological state characterized by persistent concern about falling, and has been variably defined as worry about being unable to perform usual activities without falling or a lack of confidence in maintaining balance during everyday activities [18]. Importantly, an increase in FOF does not necessarily require a preceding fall event [19]. FOF has been reported to be more prevalent and severe in HD patients than in age-matched community-dwelling older adults [20]. Elevated FOF may contribute to a vicious cycle by promoting reduced physical activity and the progression of physical frailty, thereby increasing fall risk in dialysis patients [6] (Figure 2). This is also the case in PD patients [3]. Accordingly, assessment and intervention strategies that incorporate both psychological and physical domains are essential.

The Falls Efficacy Scale–International (FES-I) is a commonly used instrument for assessing FOF. Total scores range from 16 to 64, with higher scores indicating greater FOF in daily life [21]. In dialysis patients, a cut-off value of 37.5 points on the FES-I has been reported to discriminate between fallers and non-fallers [6]. This cutoff value is based on a specific research population (Japanese HD patients), and caution is needed when applying it due to differences in language, culture, and dialysis administration methods.

### Do

Explicitly position FOF as a key component of fall risk assessment and evaluate it regularly using validated questionnaires. For patients with elevated FOF, comprehensive interventions that include psychological support in addition to physical interventions should be considered.

### Do not

Do not dismiss FOF as a ‘personality trait’ or excessive anxiety. It is also inappropriate to omit FOF assessment solely because physical function appears to be preserved.

### Pitfalls/limitations

FOF assessment tools and proposed cut-off values are not fully standardized, and results should be interpreted with caution. Moreover, some patients may not experience improvements in FOF through exercise-based interventions alone.

### Tip 4: Prioritize muscle strength and physical function over muscle mass

In HD patients, neither the diagnosis of sarcopenia nor appendicular skeletal muscle mass has been consistently associated with falls [5]. In contrast, declines in muscle strength and physical function have been consistently linked to fall risk [5, 8]. Although associations between falls and measures of muscle mass, such as quadriceps muscle thickness [22] and calf circumference [23], have been reported, clinical fall risk assessment should place greater emphasis on functional indicators, including muscle strength, gait speed, and balance performance, rather than muscle mass alone.

Regarding physical function assessment, gait speed has particular clinical relevance in HD patients; a walking speed of ≤ 1.0 m/s has been associated with a significantly increased risk of hospitalization and difficulty in activities of daily living [24]. In addition, a Short Physical Performance Battery (SPPB) score of ≤ 9 comprising standing balance, 4-m usual gait speed, and the five-times chair stand test has been linked to a higher risk of falls [5]. Because the assessment of skeletal muscle mass can be influenced by body size and fluid status, careful consideration should be given to methodological consistency, including the choice of device and timing of measurement [25].

### Do

Actively utilize simple and feasible physical function measures, such as handgrip strength, gait speed, sit-to-stand performance, and balance function. Balance and mobility, in particular, are closely associated with falls and should be regularly assessed as part of routine clinical care.

### Do not

Do not assume a low fall risk solely because skeletal muscle mass appears preserved. Likewise, do not base intervention decisions only on the presence or absence of a sarcopenia diagnosis.

### Pitfalls/limitations

Physical function assessments are sensitive to testing environments and examiner expertise. Moreover, many commonly used cut-off values are derived from community-dwelling older adults and may not be specific to dialysis patients, warranting cautious interpretation.

### Tip 5: Frailty integrates multiple fall-related risk factors

Frailty is a multidimensional construct encompassing physical, psychological, and social domains [26], and provides a valuable framework for comprehensively capturing fall risk in patients undergoing dialysis. These patients are particularly vulnerable to frailty due to a range of contributing factors, including uremic symptoms, long-term malnutrition, increased catabolism, chronic inflammation, multiple comorbidities, metabolic acidosis, physical inactivity, and amino acid loss associated with dialysis therapy [27]. The overall prevalence of frailty in HD patients has been reported to be 39.6%, with approximately 53.2% among those aged ≥ 60 years and 29.2% among those aged < 60 years [28]. Furthermore, a negative correlation has been observed between frailty and femoral neck bone mineral density in PD patients [29].

Frailty is most commonly assessed using the Fried frailty phenotype, which evaluates five physical components: slowness (reduced gait speed), low physical activity, exhaustion, shrinking (unintentional weight loss > 5% over the past year), and weakness (reduced handgrip strength). Patients meeting three or more of these criteria are classified as frail, those meeting one or two as prefrail, and those meeting none as robust [30, 31]. Alternatively, the Kihon Checklist, a brief self-administered yes/no questionnaire widely used in Japan, consists of 25 items across seven domains: instrumental activities of daily living, physical function, nutrition, oral function, social withdrawal, cognitive function, and depressive mood, allowing for a multidimensional assessment [32]. Each item is scored dichotomously (0 or 1 point), with higher total scores indicating a greater need for support or care. Frailty is defined as a total score of ≥ 8 points, prefrailty as 4–7, and robustness as 0–3[32]. Using either assessment approach, frailty dialysis patients have an approximately three- to sixfold higher fall risk compared with robust individuals [4, 11]. Accordingly, frailty assessment is essential for early identification of patients at high risk of falls.

On the other hand, compatibility between frailty assessment tools is low, and classification may differ, even for the same patient, depending on the assessment method [33]. Therefore, it is important to continue using the same tool when conducting longitudinal assessments or inter-institutional comparisons. In clinical practice, the Clinical Frailty Scale (CFS) is practical from the standpoint of simplicity and reproducibility [34]. The CFS is the most widely used scale for assessing frailty in dialysis patients [35]. It is a clinically-based frailty assessment tool that evaluates specific areas such as comorbidities, function, and cognition, and generates a frailty score ranging from 1 (very healthy) to 9 (terminal) [34]. Assessment can be performed in a short time, and a higher score indicates more severe frailty. Furthermore, the most common report uses a cutoff value of 5 points for frailty [34].

### Do

Use validated frailty assessment tools to regularly monitor frailty status. Early intervention at the prefrail or even robust stage rather than waiting until frailty is established may help reduce subsequent fall risk.

### Do not

Do not regard frailty as an irreversible condition and forgo assessment or intervention. In addition, frailty should not be evaluated solely on physical aspects; psychological and social domains must also be considered.

### Pitfalls/limitations

Frailty prevalence varies substantially depending on the assessment tool used, which should be taken into account when interpreting results. In addition, caution is required in order to avoid overestimating frailty during transient periods following acute illness or hospitalization.

### Tip 6: Conduct a comprehensive assessment, including cognitive and sensory functions and the living environment

Falls are multifactorial events involving not only physical factors such as decreased muscle strength and physical function, but also psychological factors, such as cognitive decline, visual impairment, peripheral neuropathy, and depression, as well as living environment factors [12]. These non-physical factors are frequently observed in dialysis patients in particular, due to uremia, diabetes, aging, and the effects of medications [36–38].

Cognitive decline and delirium reduce an individual’s ability to avoid danger while walking through decreased attention and executive function, increasing the risk of falls [12]. Visual impairment also makes it difficult to recognize steps and obstacles, and is an important factor involved in falls both indoors and outdoors [12]. Furthermore, peripheral neuropathy such as diabetic neuropathy impairs balance control through decreased sensory input, increasing the risk of falls [39]. In addition, both depression and use of antidepressants increase the risk of falls [40]. Finally, environmental factors such as ascension or descension of stairs/steps, insufficient lighting, lack of handrails, and slippery floors in the home and institutional environment are also important modifiable risk factors [12]. In fall prevention in dialysis patients, it is essential to take an integrated approach that assesses these cognitive, sensory, psychological, and environmental factors in addition to physical function assessment.

### Do

It is important to systematically assess cognitive function (e.g., simple cognitive function tests), visual function, presence or absence of peripheral neuropathy, and depressive state. Furthermore, it is desirable to gather information from family members and caregivers as needed, and to evaluate the home environment (e.g., presence of steps, lighting, handrails). It is also necessary to collaborate with multiple professionals and intervene in modifiable factors.

### Do not

Fall risk should not be judged solely on physical function assessment. Furthermore, cognitive decline and depression should not be overlooked as being simply due to aging, nor should environmental factors be omitted from the assessment.

### Pitfalls/limitations

Assessing these non-physical factors may require assessment tools and professional involvement, and their feasibility varies from facility to facility. Furthermore, the causal relationship between each factor and falls is complex, making it difficult to explain the risk with a single factor alone. Therefore, comprehensive and individualized assessment and interpretation are required.

### Tip 7: Do not overlook dialysis-related hemodynamic changes

IDH is an independent risk factor for falls in patients undergoing dialysis [8]. Falls occurring after dialysis, often preceded by dizziness or lightheadedness, have also been associated with an increased risk of fractures [5]. Accordingly, particular caution is warranted during dialysis sessions when blood pressure declines or therapeutic interventions for hypotension are required. In addition, rapid fluid removal during dialysis can induce marked intravascular volume shifts, leading to blood pressure instability and severe post-dialysis fatigue, which may further increase fall risk [41].

Cardiovascular autonomic dysfunction, commonly observed in dialysis patients [42], contributes to impaired cardiovascular regulation. Orthostatic hypotension and baroreflex dysfunction during postural changes have been associated with an increased frequency of falls in this population [43, 44]. Moreover, dialysis-related hemodynamic instability may also cause transient cerebral hypoperfusion, provoking dizziness or unsteadiness and further increasing fall risk [45, 46]. Taken together, these findings underscore the need for comprehensive monitoring and management strategies that include evaluation of blood pressure variability, optimization of dialysis prescriptions, and attention to post-dialysis activity environments.

IDH is commonly defined as a decrease in systolic blood pressure of ≥ 20 mmHg or a decrease in mean arterial pressure of ≥ 10 mmHg during dialysis, accompanied by hypotension-related symptoms [47]. Additionally, a nadir systolic blood pressure (SBP) < 100 mmHg [48] or a nadir diastolic blood pressure (DBP) < 50 mmHg [49] has been associated with increased mortality risk. Patients who experience recurrent hypotensive episodes over the past year have also been reported to have a poor prognosis [50]. Recent studies have shown that both a nadir SBP < 90 mmHg during dialysis, along with hypotensive event interventions, and a nadir DBP < 50 mmHg are associated with severe falls [51].

### Do

Systematically assess blood pressure fluctuations and subjective symptoms during and after dialysis, and evaluate their potential associations with falls. When appropriate, adjust dialysis conditions and implement measures to ensure a safer post-dialysis activity environment.

### Do not

Do not treat falls as merely accidental events without evaluating underlying hemodynamic factors. Likewise, avoid underestimating the risks associated with patient activities immediately after dialysis.

### Pitfalls/limitations

Blood pressure measurements alone may not fully capture underlying autonomic dysfunction. Caution is therefore required when interpreting the causal relationships between hemodynamic instability and falls.

### Tip 8: Exercise therapy is a core intervention for fall prevention

In patients undergoing dialysis, declines in muscle strength and physical function, as well as frailty, are closely associated with fall risk. Exercise therapy, therefore, plays a central role in directly targeting these modifiable factors. Exercise interventions in dialysis patients have the potential to significantly reduce fall incidence, although the current evidence remains limited [52]. Balance training and resistance exercise are generally considered effective for fall prevention [53]; however, careful attention to safety is essential when implementing these programs.

Exercise therapy during dialysis sessions or on non-dialysis days, as well as the promotion of physical activity in daily life, may contribute to the maintenance and improvement of physical function in dialysis patients [54, 55]. Although intradialytic exercise appears to have smaller effects on exercise tolerance than supervised exercise performed on non-dialysis days, it may be particularly beneficial for long-term adherence due to its lower dropout rates [56]. Importantly, intradialytic exercise has also been shown not to exacerbate hemodynamic instability during dialysis [57]. However, reduced baseline gait speed, advanced age, elevated inflammatory markers, and lower dialysis dose have been reported as determinants of dropout from intradialytic exercise programs [58], indicating that individualized adjustments to exercise intensity and goal setting may be necessary.

In recent years, there has been growing interest in home-based exercise programs. A systematic review and meta-analysis of randomized controlled trials in dialysis patients has shown that home-based exercise programs significantly improve walking ability (6-minute walk distance) and aerobic capacity (maximum oxygen uptake) [59]. Furthermore, no significant difference was found regarding the improvement of physical function when compared to exercise performed during dialysis, and it has been reported that home exercise has effects comparable to those of exercise performed during dialysis [59]. These results suggest that home-based exercise is independent of the resources of dialysis facilities, contributes to increased patient autonomy, and allows for the selection of exercise programs according to patient preferences and facility conditions [59]. Nevertheless, exercise programs are beneficial to dialysis patients regardless of where they are performed.

While knowledge regarding exercise therapy for PD patients remains limited, a randomized controlled trial of home-based exercise reported improved exercise tolerance (shuttle walking time) in an exercise group [60]. Furthermore, to the best of our knowledge, no serious adverse events (death, hospitalization, etc.) attributable to exercise intervention in PD patients have yet been reported [61]. Since home-based exercise therapy is fundamental for PD patients, maintaining exercise adherence is extremely important; therefore, continuous intervention by a multidisciplinary team is crucial.

### Do

Support sustainable exercise and physical activity in a manner tailored to patients’ physical function, dialysis conditions, and living environments. Intradialytic exercise, in particular, can be safely continued under medical supervision and may facilitate long-term adherence.

### Do not

Do not uniformly prescribe high-intensity exercise or allow exercise to be performed without appropriate safety management. Moreover, given the limited evidence directly linking exercise therapy to future fall reduction, exercise alone should not be overestimated as a comprehensive preventive strategy.

### Pitfalls/limitations

Ensuring safety during the initial phase of intervention can be challenging in patients at high risk of falls. In addition, the effectiveness of exercise interventions is highly dependent on long-term adherence.

### Tip 9: Avoid one-time assessments—reassess and adapt over time

**Figure 3:**
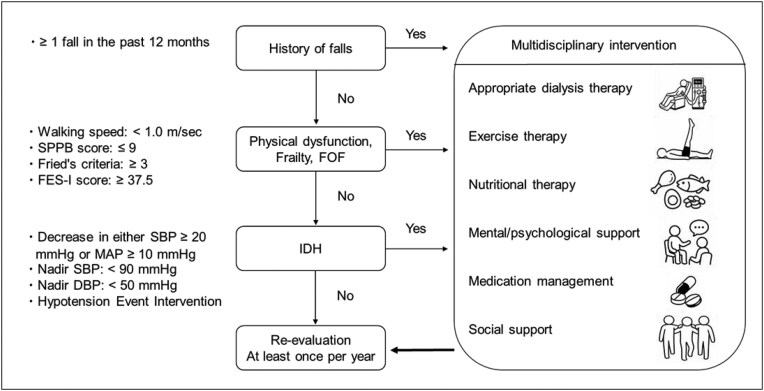
Flowchart of fall risk assessment and multidisciplinary intervention in patients undergoing dialysis. This flowchart guides sequential evaluation of key risk domains and emphasizes interprofessional collaboration to address identified factors. It is designed to support clinical decision-making in routine practice rather than prescribe rigid protocols.

Physical function and fall risk in HD patients change over time as dialysis duration increases [62]. Physical function should be assessed at least once a year before prescribing physical activity or exercise programs, with prescriptions adjusted individually, and reassessed every three or six months during the intervention to monitor improvement [63]. Furthermore, physical activity and exercise programs should begin at a low intensity and be gradually increased according to the patient’s tolerance [63]. Because fall risk fluctuates dynamically in response to factors such as progression of frailty or comorbid conditions, modifications in dialysis prescriptions, and changes in physical activity levels, regular reassessment and adjustment of intervention strategies are essential (Figure 3) [12]. In particular, since a history of hospitalization in the past year is associated with worsening frailty in HD patients [64], assessment of physical function at discharge is important. Cut-off values used to define elevated fall risk should therefore be regarded as flexible reference points rather than fixed diagnostic thresholds. Depending on changes in clinical status, dialysis-related factors, and responses to interventions, these values may require periodic reevaluation and adjustment.

### Do

Integrate regular reassessment into routine clinical workflows and modify interventions in accordance with changes in patient status.

### Do not

Do not make static judgments about fall risk based solely on the initial assessment. Likewise, avoid omitting reassessment following changes in dialysis conditions.

### Pitfalls/limitations

There is no clear consensus on the optimal frequency of reassessment, and its practical implementation may depend on institutional resources and clinical infrastructure.

### Tip 10: Sustained, multidisciplinary approaches are essential

Falls in dialysis patients are multifactorial events in which general fall-related risk factors are compounded by dialysis-specific treatment-related factors. In addition, medication management and social support should be considered integral components of a comprehensive, multidisciplinary fall-prevention strategy in dialysis care [65, 66]. In particular, medication use, including polypharmacy [67], psychoactive and anticholinergic drugs (antidepressants, anxiolytics, hypnotics, antipsychotics), cardiovascular drugs (antihypertensives, beta-blockers, diuretics, digoxin), and analgesics (NSAIDs, antiepileptics, opioids), is associated with falls via sedation, orthostatic hypotension, or other mechanisms [68].

**Figure 4:**
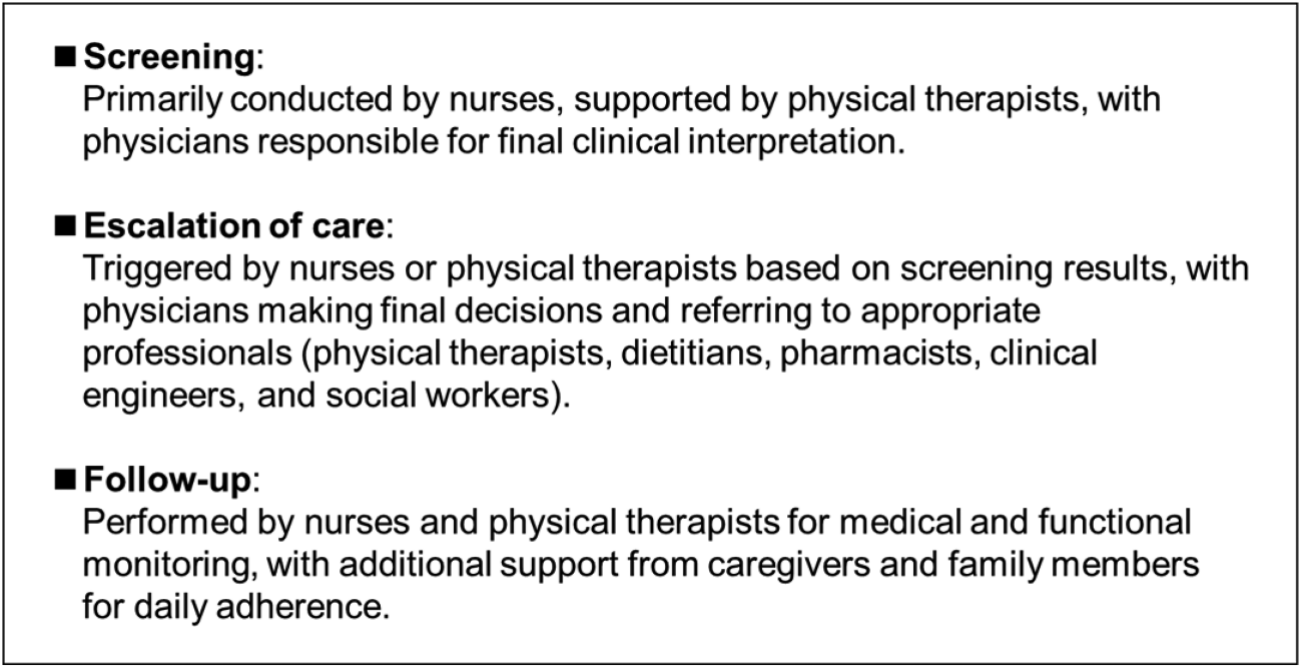
Multidisciplinary framework for exercise therapy implementation targeting fall prevention in hemodialysis patients. Fall risk is screened by nurses with support from physical therapists and interpreted by physicians. When risk is identified, care is escalated with physician-led decisions and referral to appropriate professionals. Follow-up by nurses and physical therapists monitors outcomes, with support from caregivers and family.

Accordingly, effective fall prevention requires collaboration not only among healthcare professionals—such as physicians, nurses, physical therapists, registered dietitians, clinical engineers, pharmacists, and social workers—but also with caregivers and family members (Figure 3). Within this framework, identifying individual fall risk factors and implementing comprehensive, sustained, multidisciplinary interventions are crucial. (Figure 4) [12, 65, 66].

### Do

Establish coordinated multidisciplinary collaboration with clear role definitions and effective information sharing among team members.

### Do not

Do not delegate fall prevention to a single profession, nor discontinue interventions solely based on short-term outcomes.

### Pitfalls/limitations

The implementation of multidisciplinary collaboration varies across institutions, and supporting evidence remains limited.

## CONCLUSION

Falls in patients undergoing dialysis are multifactorial events resulting from interactions between general fall risk factors and dialysis-specific conditions, including hemodynamic instability. The ten tips presented in this review provide a structured and clinically applicable framework for assessing fall risk and implementing preventive strategies in dialysis settings. A key principle is that fall risk should be viewed as dynamic rather than static, requiring regular reassessment and adjustment of interventions in response to changes in clinical status and dialysis-related factors. Exercise-based interventions represent an important component of fall prevention but should be integrated into a comprehensive, multidisciplinary approach involving healthcare professionals, caregivers, and social support systems. Future studies are needed to establish standardized assessment protocols and to evaluate the effectiveness of longitudinal, multidisciplinary fall prevention programs. Incorporating these principles into routine dialysis care may contribute to improved safety, functional independence, and quality of life in this vulnerable population. It is noteworthy that, although the framework presented in this review is intended to support clinical decision-making, its effectiveness needs to be verified by future research. Not all interventions are guaranteed to uniformly reduce falls.

## Data Availability

No new data were generated or analyzed in support of this research.
